# Exosome miR-4738-3p-mediated regulation of *COL1A2* through the NF-κB and inflammation signaling pathway alleviates osteoarthritis low-grade inflammation symptoms

**DOI:** 10.17305/bb.2023.9921

**Published:** 2024-06-01

**Authors:** Jun Xu, Kaifeng Zhou, Huijie Gu, Yiming Zhang, Liang Wu, Chong Bian, Zhongyue Huang, Guangnan Chen, Xiangyang Cheng, Xiaofan Yin

**Affiliations:** 1 Department of Orthopaedics, Minhang Hospital, Fudan University, Shanghai, China

**Keywords:** Exosome, microRNA (miR)-4739-3p, collagen type I alpha 2 chain (*COL1A2*), nuclear factor kappa B (NF-κB) signaling pathway, osteoarthritis (OA)

## Abstract

This study aimed to elucidate the roles of microRNA (miR)-4738-3p and the collagen type I alpha 2 chain (*COL1A2*) gene in the pathogenesis of osteoarthritis (OA) through bioinformatics analysis and cellular assays. The GSE55235 dataset was analyzed using the weighted gene co-expression network analysis (WGCNA) method to identify gene modules associated with OA. Key overlapping genes were identified from these modules and the GSE55235-differentially expressed genes (DEGs). The expression levels of selected genes were determined in C28/I2 cells using the quantitative real-time polymerase chain reaction (qRT-PCR). The interaction between miR-4738-3p and *COL1A2* was examined in the context of interleukin 1 beta (IL-1β) induction. Exosome characterization was achieved through transmission electron microscopy (TEM), western blotting (WB), and other analyses. The study also investigated the functional relevance of miR-4738-3p in OA pathology through various molecular and cellular assays. Our findings revealed that the green module exhibited a strong correlation with the OA phenotype in the GSE55235 dataset, with *COL1A2* emerging as a hub gene and miR-4738-3p as its key downstream target. IL-1β induction suggested that *COL1A2* is involved in inflammation and apoptosis, while miR-4738-3p appeared to play an antagonistic role. The analysis of exosomes underscored the significance of miR-4738-3p in cellular communication, with an enhanced level of exo-miR-4738-3p antagonizing IL-1β-induced inflammation and promoting cell survival. Conversely, a reduction in exo-miR-4738-3p led to increased cell damage. This study established a clear regulatory relationship between miR-4738-3p and *COL1A2*, with the nuclear factor kappa B (NF-κB) signaling pathway playing a central role in this regulation. The miR-4738-3p significantly influences the OA-associated inflammation, primarily through the modulation of *COL1A2* and the NF-κB pathway. Therefore, targeting miR-4738-3p offers a potential therapeutic approach for OA, with exosome miR-4738-3p presenting a promising strategy.

## Introduction

Osteoarthritis (OA) is a chronic degenerative joint disease affecting over 500 million people worldwide. The traditional view that OA is caused primarily by the progressive wear and tear of articular cartilage has changed. It is now more accurately characterized as a chronic total joint disease. This disease initiates with biochemical and cellular alterations in the synovial tissues of the joints, eventually leading to histologic and structural changes within the joint. These changes ultimately result in total joint dysfunction [1]. Globally, millions suffer from OA, making it a leading cause of disability among the aging population [2, 3]. This increasing prevalence of OA is partly attributable to a rise in risk factors for OA, including obesity, physical inactivity, and joint injuries. Joint pain associated with OA not only causes functional limitations but also leads to decreased sleep quality, fatigue, mood disturbances, and even a loss of independence. Statistically, a majority of people with OA (59%–87%) also suffer from at least one other chronic disease, particularly cardiometabolic disease. Symptomatic OA can impair the ability of patients with cardiometabolic diseases to exercise and manage weight, thereby increasing the risk of adverse outcomes [4]. Clinically, it presents with symptoms, such as chronic pain, stiffness, and reduced functionality, profoundly affecting the patient’s quality of life and imposing a significant burden on healthcare systems [5]. Multiple etiological factors contribute to the onset and progression of OA, including age, genetic predisposition, joint injuries, obesity, and biomechanical imbalances [6, 7]. If left unmanaged, advanced stages of OA can lead to secondary complications, such as bone spurs, inflammation of the joint lining, and even joint deformities [8]. While various pharmacological and surgical interventions are available, they typically provide symptomatic relief rather than disease modification [9]. Current research in addressing OA is focused on promising strategies like gene therapy, which targets specific molecular pathways to potentially halt or reverse cartilage degradation, although these approaches are still in the early stages of clinical application [10, 11]. This study concentrates on the emerging gene therapies for OA, evaluating their efficacy and potential implications for future treatment modalities.

Building on the perspective that gene therapy is a promising approach for managing OA, it becomes pivotal to understand the genetic factors in the disease pathway. The collagen type I alpha 2 chain (*COL1A2)* gene, responsible for encoding the alpha 2 chain of type I collagen, is of particular interest [12]. Mutations in *COL1A2* are implicated in various disorders, notably osteogenesis imperfecta types III and IV, characterized by abnormal collagen leading to brittle bones and frequent fractures [13, 14]. Beyond skeletal disorders, *COL1A2* has also garnered attention for its role in inflammatory diseases, underscoring its broader relevance in pathological processes [15]. In the context of inflammation, the nuclear factor kappa B (NF-κB) signaling pathway emerges as a central player [16]. Interestingly, there is a reciprocal regulation between NF-κB and the Toll-like receptor 4 (TLR4), a TLR crucial for recognizing pathogens and initiating the innate immune response [17]. Given the established link between inflammation and OA, the interaction between this pathway and TLR4 may have profound implications for OA pathogenesis [18]. Therefore, exploring the complex relationship between NF-κB, TLR4, and OA pathogenesis may pave the way for innovative therapeutic avenues for OA management.

As we delve deeper into the intricacies of OA pathogenesis and molecular interactions, exosomes have emerged as pivotal players in the realm of intercellular communication [19, 20]. These nano-sized vesicles, secreted by various cell types including the human synovial mesenchymal stem cells (HS-MSCs), function as carriers of bioactive molecules. They play significant roles in various physiological and pathological processes, such as aiding tissue recovery and mitigating systemic inflammatory responses [21, 22]. Recent advancements underscore the potential of exosomes in pathology. For instance, MSC-derived exosomes demonstrate prospective utility in treating tendon-bone insertion injuries, underscoring their potential as gene therapy carriers, diagnostic biomarkers, and prognostic indicators [23, 24]. In the context of OA specifically, cartilage cell-derived exosomes can affect macrophage behavior, instigating synovitis and exacerbating OA through intricate pathways involving the microRNA (miR)-449a-5p and autophagy cascades, thereby modulating the interleukin 1 beta (IL-1β) release [25]. Other studies have also indicated that various miRNAs enriched within exosomes, such as miR-124, miR-206, and miR-140-5p, are instrumental in OA progression [26, 27], suggesting a broad connection between numerous miRNAs in exosomes and OA. However, the specific role and implications of miR-4738-3p in OA remain elusive and warrant further elucidation.

In light of the intricate relationship between gene expression patterns and OA progression, the present study endeavored to elucidate the molecular pathways and key contributors orchestrating the OA phenotype, utilizing the GSE55235 dataset. Central to our groundbreaking findings is the pivotal role of miR-4738-3p and *COL1A2* in regulating OA outcomes. We sought to analyze the function of the green module, which is closely associated with the OA phenotype, and to deeply investigate the complex interactions among HS-MSC-exosome-enriched miR-4738-3p, *COL1A2*, and the TLR4/NF-κB signaling pathway. Employing a combination of bioinformatic analysis and in vitro experimental validations, this research seeks to shed light on novel molecular markers potent for OA therapeutic intervention, thereby adding a new dimension to the comprehensive understanding of OA pathogenesis.

## Materials and methods

### Screening of differentially expressed genes (DEGs) in the GSE55235 dataset

The GSE55235 dataset, pertaining to OA, was retrieved from the Gene Expression Omnibus (GEO) database, accessible at https://www.ncbi.nlm.nih.gov/geo/. This dataset encompasses samples from ten healthy synovial tissues, ten osteoarthritic synovial tissues, and ten rheumatoid arthritis synovial tissues. For this study, we selected the ten healthy synovial tissue samples as the control group and the ten osteoarthritic synovial tissue samples as the OA group. We conducted a differential gene expression analysis using the GEO2R tool. The DEGs were identified based on a fold change (FC) of either greater than 2 or less than 0.5, with a significance threshold set at *P* < 0.05.

### Weighted gene co-expression network analysis (WGCNA)

For a comprehensive analysis of the entire gene set from both the control and OA samples within the GSE55235 dataset, the WGCNA method was employed. The optimal soft-thresholding power was ascertained by evaluating the scale-free topology fit index across varying powers. A sample dendrogram, accompanied by a trait heatmap, was constructed to visualize sample clustering and identify potential outliers. With the chosen soft-thresholding power, an adjacency matrix was created, which was then transformed into a topological overlap matrix (TOM). This matrix informed the generation of a gene dissimilarity measure (1-TOM), facilitating hierarchical gene clustering and the identification of gene modules through dynamic tree cutting. Subsequent visual analysis was conducted to explore the relationships between different gene modules. Additionally, modular trait relationships were examined by associating module trait genes with the control and OA groups, thereby identifying the key module that exhibited the strongest association with phenotypic traits in both groups.

### Expression analysis of key overlapping genes

After identifying the green module as the crucial component, we utilized the “VennDiagram” package in R to conduct a cross-analysis between this key module and the DEGs of GSE55235. This analysis was crucial for identifying overlapping genes. Based on the proteins encoded by these overlapping genes, a protein–protein interaction (PPI) analysis was conducted using the Search Tool for the Retrieval of Interacting Genes (STRING; https://string-db.org/) database, in conjunction with the Cytoscape software. Within Cytoscape, specific algorithms from the Cytohubba plug-in were employed, namely, the Molecular Complex Detection (MOCDE), Maximal Clique Centrality (MCC), and Dense Module based on Node Connectivity (DMNC), to visualize key modules in the PPI network. Subsequently, an intersection analysis was performed on the three identified key modules to identify key overlapping genes. Finally, the expression levels of these key overlapping genes were assessed in the OA and control samples within the GSE55235 dataset.

### Analysis of the key miRNA and its correlation with *COL1A2*

We identified 259 and 117 miRNAs associated with *COL1A2* from the TargetScan (https://www.targetscan.org) and the microRNA Target Prediction Database (miRDB; http://www.mirdb.org/) databases, respectively. Concurrently, ten miRNAs were retrieved from the differentially expressed (DE) miRNAs in the GSE55235 dataset. An intersection analysis was then conducted across miRNAs from both the TargetScan and miRDB databases, and the ten DE miRNAs from GSE55235, leading to the identification of a key miRNA of interest. Subsequent steps involved examining the expression levels of this miRNA within the OA and control samples from the GSE55235 dataset. To further investigate the relationship between the identified miRNA and *COL1A2*, a correlation analysis was executed using the Gene Expression Profiling Interactive Analysis (GEPIA) database (http://gepia.cancer-pku.cn/). This analysis aimed to uncover potential regulatory interactions between the miRNA and *COL1A2*.

### Cell culture and IL-1β treatment

C28/I2 chondrocytes and HS-MSCs were obtained from Shanghai YS Industrial Co., Ltd. These cells were cultured under standard conditions, which include a temperature of 37 ^∘^C and a 5% CO_2_ atmosphere, in a Roswell Park Memorial Institute 1640 (RPMI1640) medium. This medium was supplemented with 10% fetal bovine serum (FBS), 100 U/mL penicillin, and 100 µg/mL streptomycin. To establish an in vitro OA model, the cells were treated with various concentrations of IL-1β (specifically 2.5, 5, and 10 ng/mL) for a specified duration of 24 h. Additionally, to evaluate the effect of the NF-κB pathway in this context, cells were pre-treated with BAY 11-7082, an inhibitor of NF-κB activation, for 2 h prior to the IL-1β stimulation.

### Transfection assay

Transfection procedures were carried out using Lipofectamine 2000, adhering strictly to the manufacturer’s instructions. The chondrocytes were subjected to various transfection regimes based on experimental design. These regimens included the use of mimics (negative control [NC] and miR-4738-3p), inhibitors (NC and miR-4738-3p), a small interfering RNA (siRNA) targeting *COL1A2* (si-*COL1A2*), and a non-targeting control siRNA (si-NC) (GenePharma, Shanghai, China). Additionally, we utilized overexpression vectors (OE-*COL1A2*) and an empty vector control (pcDNA). In specific experiments involving HS-MSCs exosomes, the cells were exposed to inhibitors encapsulated within exosomes, designated as exo-NC-inhibitor and exo-miR-4738-3p inhibitor. Following the transfection, the cells were cultured for an additional 48 h prior to conducting further experimental procedures or data collection.

### Enzyme-linked immunosorbent assay (ELISA) for IL-1β determination

IL-1β levels in the supernatants from IL-1β-treated C28/I2 chondrocytes (using basal medium) were quantified using a human IL-1β ELISA kit (Abcam American, ab212166). The procedure began with the addition of collected supernatants and standards into wells that were pre-coated with an anti-IL-1β antibody. Following incubation and subsequent washing steps, a biotinylated anti-IL-1β antibody was applied. This was followed by the addition of horseradish peroxidase (HRP)-conjugated streptavidin. After another incubation and washing, a substrate reactive to HRP was introduced to the wells, initiating a colorimetric reaction that was terminated with a stop solution. The intensity of the color developed, which correlates with the IL-1β concentration, was measured at 450 nm using an ELISA reader (BioTek Instruments, Inc., Winooski, VT, USA). The IL-1β concentration in the samples was determined by comparing their optical densities to a standard curve generated from the provided standards.

### Quantitative real-time polymerase chain reaction (qRT-PCR)

Total RNA was extracted from the C28/I2 chondrocytes using TRIzol reagent (Invitrogen, Carlsbad, CA, USA). The synthesis of complementary DNA (cDNA) was then performed using a reverse transcription kit (Thermo Fisher Scientific, Waltham, MA, USA), adhering strictly to the manufacturer’s protocol. qRT-PCR was conducted using a SYBR Green Master Mix (Bio-Rad Laboratories, Hercules, CA, USA). Specific primers targeting miR-4738-3p, *COL1A1, COL1A2, COL3A1, COL5A1, COL5A2,* cyclooxygenase 2 (*COX2*), inducible nitric oxide synthase (*INOS*), matrix metallopeptidase 13 (*MMP13*), collagen 2, cleaved (c)-Caspase3, B-cell lymphoma 2 protein (*Bcl-2*), and the internal controls (glyceraldehyde 3-phosphate dehydrogenase [*GAPDH*], U6 small nuclear RNA [U6]) were employed. Relative gene expression levels were calculated using the 2^-ΔΔCt^ method, with normalization against the internal control. The sequences of the primers used in this study are detailed in Table S1.

### Western blotting (WB)

Proteins were extracted from both treated and control C28/I2 chondrocytes using the radioimmunoprecipitation assay (RIPA) buffer, which included protease and phosphatase inhibitors. Protein concentrations were determined using the bicinchoninic acid (BCA) assay. Equal amounts of protein were then subjected to separation on 10%–12% sodium dodecyl sulfate-polyacrylamide gel electrophoresis (SDS-PAGE) and subsequently transferred onto polyvinylidene difluoride (PVDF) membranes (Millipore). For blocking, the membranes were incubated in 5% bovine serum albumin (BSA) in tris-buffered saline with tween (TBST) buffer for 1 h, followed by overnight incubation at 4 ^∘^C with primary antibodies targeting COL1A2, COX2, INOS, MMP13, collagen 2, TLR4, NF-κB, phosphorylated NF-κB (p-NF-κB), c-Caspase3, Bcl-2, cluster of differentiation 9 (CD9), CD81, and heat shock protein 70 (HSP70), all diluted at 1:1000. Post-primary antibody incubation, the membranes were exposed to HRP-conjugated secondary antibodies at a 1:5000 dilution for 1 h. Bands were detected using the enhanced chemiluminescence (ECL) system (Amersham) and their intensities were quantified by densitometry. For normalization, membranes were stripped and re-probed with an anti-β-actin antibody as loading controls. Band intensity analysis was performed using ImageJ software.

### Flow cytometry analysis

After a 24-h treatment with IL-1β, the chondrocytes were harvested and washed with cold phosphate-buffered saline (PBS). Subsequently, they were stained using Annexin V-fluorescein isothiocyanate (FITC) and propidium iodide (PI) as per the protocol provided by Annexin V-FITC Apoptosis Detection Kit (Keygen Company, Nanjing, China). Following a 20-min incubation period, the cells were analyzed using a flow cytometer (BD FACSCANTO II, BD Biosciences, San Jose, CA, USA). Cells in the early stages of apoptosis were identified as being positive for Annexin V-FITC and negative for PI, whereas cells in the later stages of apoptosis exhibited positivity for both Annexin V-FITC and PI.

### Transmission electron microscopy (TEM)

Exosomes were isolated from the conditioned medium of HS-MSCs. The initial step involved removing cells and debris through sequential centrifugation, first at 300 × *g* for 10 min, followed by 2000 × *g* for 20 min. Subsequently, the supernatant was subjected to ultracentrifugation at 100,000 × *g* for 70 min to precipitate the exosomes. The resultant exosome pellet was then resuspended in PBS for downstream applications. For morphological analysis using TEM, exosome suspensions were applied onto formvar carbon-coated grids. The samples were stained with 2% uranyl acetate and visualized under a TEM. Specifically, 20 µL of the exosome suspension was placed on the grid and incubated for 5 min at room temperature. After incubation, the excess solution was removed and the grid was dried with filter paper for 30 min. Subsequently, a negative staining step was performed using an equal amount of 10% uric acid ketonate for 5 min. The size distribution and concentration of the isolated exosomes were ascertained using a nanoparticle tracking analyzer (N30E, NanoFCM, Xiamen, China).

### Dual-luciferase reporter assay

To investigate the interaction between miR-4738-3p and the 3′-untranslated region (3′-UTR) of *COL1A2*, a dual-luciferase reporter assay was employed. The 3′-UTR of *COL1A2*, containing the predicted binding sites for miR-4738-3p, was cloned into a luciferase reporter vector. Chondrocytes, at 70%–80% confluence, were co-transfected with this reporter and either miR-4738-3p mimics or inhibitors. Luciferase activity was assessed 48-h post-transfection using a dual-luciferase assay kit (Beyotime Biotechnology). In this assay, firefly luciferase activity was used as an internal control to normalize the results.

### Ethical statement

This study received approval from the Ethics Review Board of Minhang Hospital, Fudan University.

### Statistical analysis

All experiments in this study were conducted in triplicate. Data are presented as mean ± standard deviation (SD). To evaluate differences among groups, a one-way analysis of variance (ANOVA) was employed. Pairwise comparisons were subsequently carried out using Tukey’s post-hoc test to determine specific differences between groups. The level of statistical significance was set at *P* < 0.05. All statistical analyses were conducted using SPSS, version 25.0.

## Results

### Identification of the green module closely related to the OA phenotype

**Figure S1.**
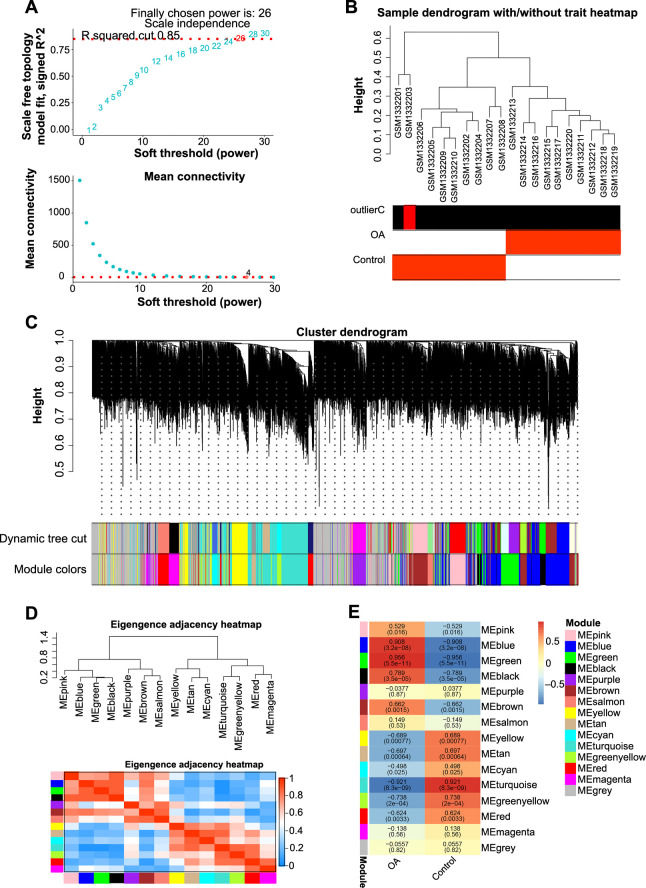
**WGCNA analysis of the GSE55235 dataset.** (A) Determination of the optimal soft-thresholding power with the panel depicting the scale-free topology fit index plotted against various soft-thresholding powers. The optimal soft-thresholding power selected for the analysis is highlighted with a vertical red line. (B) Sample dendrogram based on topological overlap, alongside a trait heatmap, illustrating the clustering patterns of the samples and highlighting potential outliers within the dataset. (C) Cluster dendrogram derived from the topological overlap matrix illustrating the hierarchical organization of genes and the formation of different modules. (D) Eigengene adjacency heatmap illustrating the relationships between different gene modules. The color gradient in the heatmap corresponds to the varying levels of adjacency between modules, with color intensity indicating the strength of these relationships. (E) Trait heatmap demonstrating the correlations between the identified gene modules and the specific traits of interest in GSE55235 samples, with corresponding correlation values and *P* values. WGCNA: Weighted gene co-expression network analysis; OA: Osteoarthritis; ME: Module eigengene.

We conducted a comprehensive gene set analysis of control and OA samples from the GSE55235 dataset employing the WGCNA method. The scale-free topology fit index pinpointed the optimal soft-thresholding power at 26 (Figure S1A). The hierarchical clustering of the 20 samples revealed different clustering patterns and potential outliers (Figure S1B). Following this, hierarchical clustering and dynamic tree cutting led to the identification of 14 gene modules, each represented by different colors (Figure S1C and S1D). Notably, among the identified gene modules, the green module emerged as the most significantly associated with the GSE55235 sample, evidenced by a high correlation coefficient of 0.956 (Figure S1E). Given the strong association with both the control and OA phenotypic traits, the green module was selected as the key module for subsequent analyses.

### Identification of five key overlapping genes

**Figure S2.**
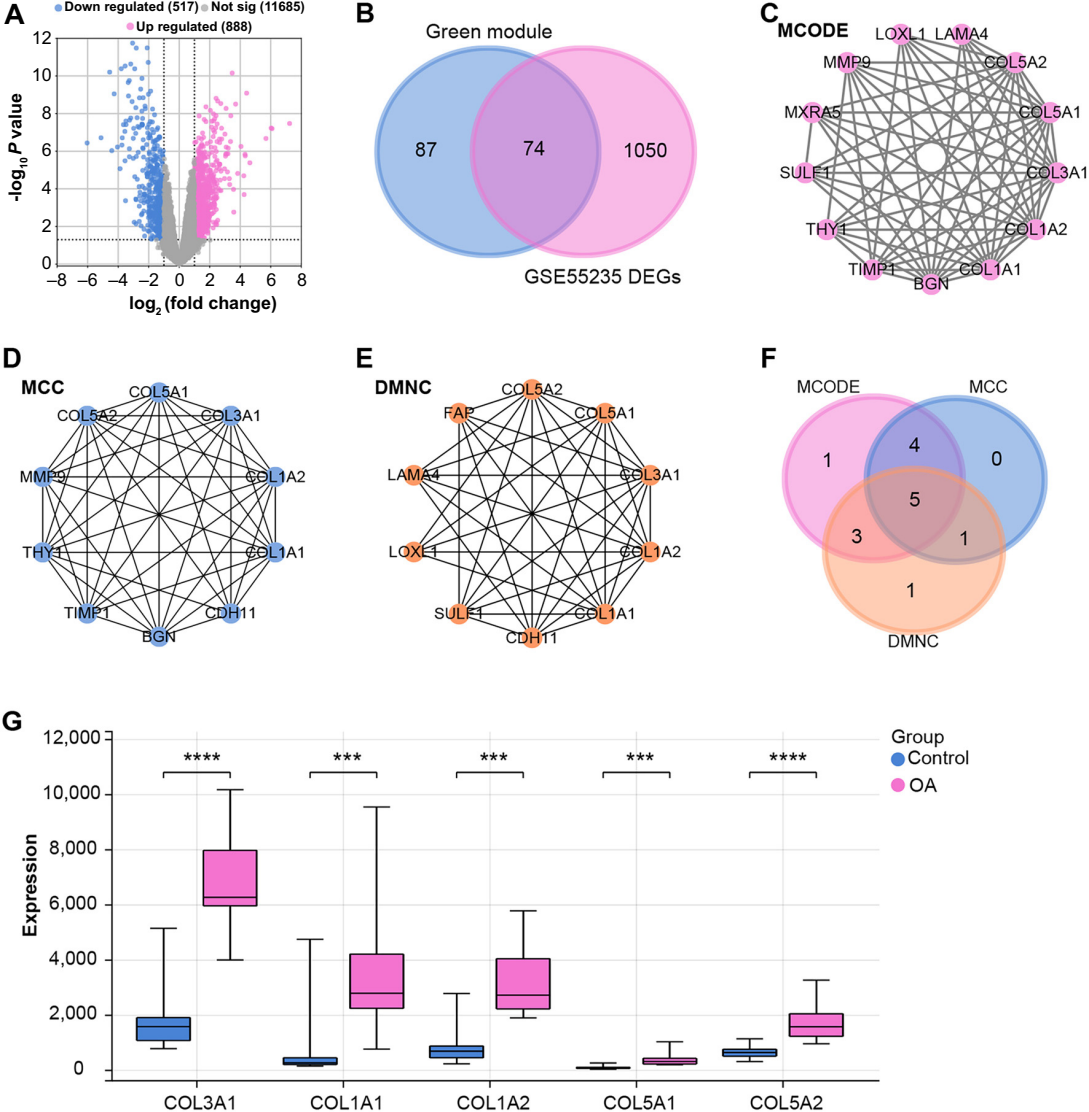
**Identification and network analysis of overlapping genes in the green module and GSE55235-DEGs.** (A) Volcano plot detailing the upregulated (red) and downregulated (blue) DEGs identified from the GSE55235 dataset, with horizontal and vertical lines indicating threshold values for significance and fold change. (B) Venn diagram highlighting the overlap of genes between the green module and GSE55235-DEGs. The overlapping region illustrates the common genes shared by both sets. (C–E) Representations of the PPI networks based on the MOCDE (C), MCC (D), and DMNC (E) algorithms, respectively, with each node in the networks representing a gene, and the edges indicating interactions between these genes. (F) Venn diagram of five key overlapping genes identified within the three sub-networks analyzed by the MOCDE, MCC, and DMNC algorithms. (G) Expression levels of five key overlapping genes (*COL3A1, COL1A1, COL1A2, COL5A1,* and *COL5A2*) in OA and control groups. The y-axis represents the expression values, with the OA group presented with pink color and the control group presented with blue color. ***P < 0.001; ****P < 0.0001. DEGs: Differentially expressed genes; PPI: Protein–protein interaction; MOCDE: Molecular Complex Detection; MCC: Maximal Clique Centrality; DMNC: Dense Module based on Node Connectivity; *LAMA4*: Laminin subunit alpha 4; *COL5A2*: Collagen type V alpha 2 chain; *COL5A1*: Collagen type V alpha 1 chain; *COL3A1*: Collagen type III alpha 1 chain; *COL1A2*: Collagen type I alpha 2 chain; *COL1A*: Collagen type I alpha 1 chain; COX2: Cyclooxygenase 2; *BGN*: Biglycan; *TIMP1*: Tissue inhibitor of metalloproteinases 1; *THY1*: Thy-1 membrane glycoprotein; *SULF1*: Sulfatase 1; *MXRA5*: Matrix-remodeling associated 5; *MMP9*: Matrix metallopeptidase 9; *LOXL1*: Lysyl oxidase-like 1; *CDH11*: Cadherin 11; *FAP*: Fibroblast activation protein.

Utilizing the GEO2R tool, a total of 888 upregulated and 517 downregulated DEGs were identified from the GSE55235 dataset (Figure S2A). A focused examination of the green module, which comprises 161 genes, in conjunction with the 1124 DEGs, led to the identification of 74 overlapping genes (Figure S2B). These overlapping genes were then subjected to a PPI network analysis. Three crucial sub-networks were identified based on MOCDE (13 nodes, 62 edges), MCC (10 nodes, 43 edges), and DMNC (10 nodes, 38 edges) algorithms (Figure S2C–S2E). Further in-depth analysis of these sub-networks revealed five pivotal overlapping genes, namely: *COL1A1*, *COL1A2*, *COL3A1*, *COL5A1*, and *COL5A2* (Figure S2F and S2G).

### Determination of *COL1A2* and its downstream target miR-4738-3p

**Figure S3.**
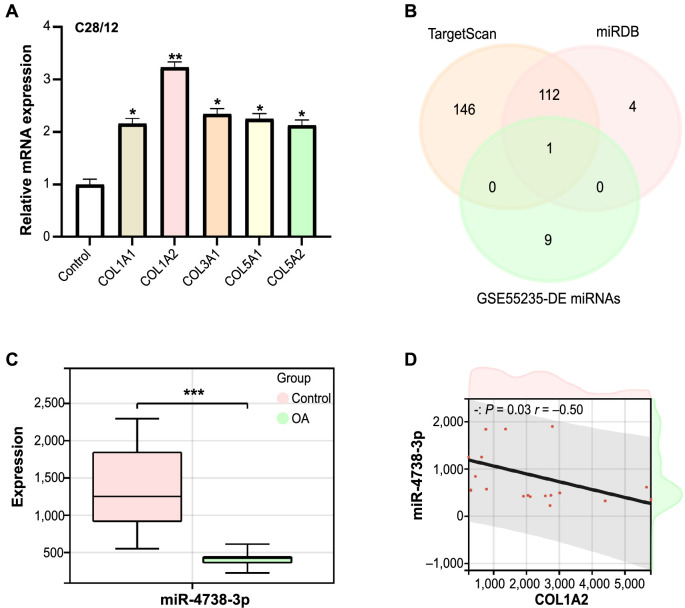
**Expression and correlation analysis of *COL1A2* and miR-4738-3p.** (A) Bar graph from the qRT-PCR analysis depicting the relative expression levels of five key overlapping genes in C28/I2 cells. The *y*-axis indicates their relative expression levels. (B) Venn diagram showing the intersection of targets identified from the TargetScan databases, miRDB databases, and the GSE55235-DE miRNAs. (C) Boxplot comparing the expression levels of miR-4738-3p in GSE55235-OA samples versus the control samples. ***P < 0.001; indicating a statistically significant difference in expression. (D) Correlation scatter plot, generated using the GEPIA database, showing a significant negative correlation between the expression of miR-4738-3p and *COL1A2*. The *P* value and correlation coefficient are shown in the upper left corner of the plot. *COL1A2*: Collagen type I alpha 2 chain; miR: MicroRNA; qRT-PCR: Quantitative real-time polymerase chain reaction; miRDB: MicroRNA Target Prediction Database; DE: Differentially expressed; miRNAs: MicroRNAs; OA: Osteoarthritis; GEPIA: Gene Expression Profiling Interactive Analysis; mRNA: Messenger RNA; *COL1A1*: Collagen type I alpha 1 chain; *COL3A1*: Collagen type III alpha 1 chain; *COL5A1*: Collagen type V alpha 1 chain; *COL5A2*: Collagen type V alpha 2 chain.

We used qRT-PCR to analyze the expression levels of five key overlapping genes in C28/I2 cells (Figure S3A). Among them, *COL1A2* exhibited the highest expression level, thereby establishing it as the central hub gene. Further investigations into the TargetScan database, miRDB database, and GSE55235-DE miRNAs led to the identification of a critical downstream target of *COL1A2*: miR-4738-3p (Figure S3B). Expression validation of miR-4738-3p indicated an upregulated expression pattern in the OA samples from the GSE55235 dataset (Figure S3C). Additionally, analysis using the GEPIA database highlighted a significant inverse correlation between miR-4738-3p and *COL1A2* expression, with a correlation coefficient of −0.5 (Figure S3D).

### IL-1β-induced chondrocyte C28/I2 injury and its regulatory effect on miR-4738-3p and *COL1A2*

**Figure 1.**
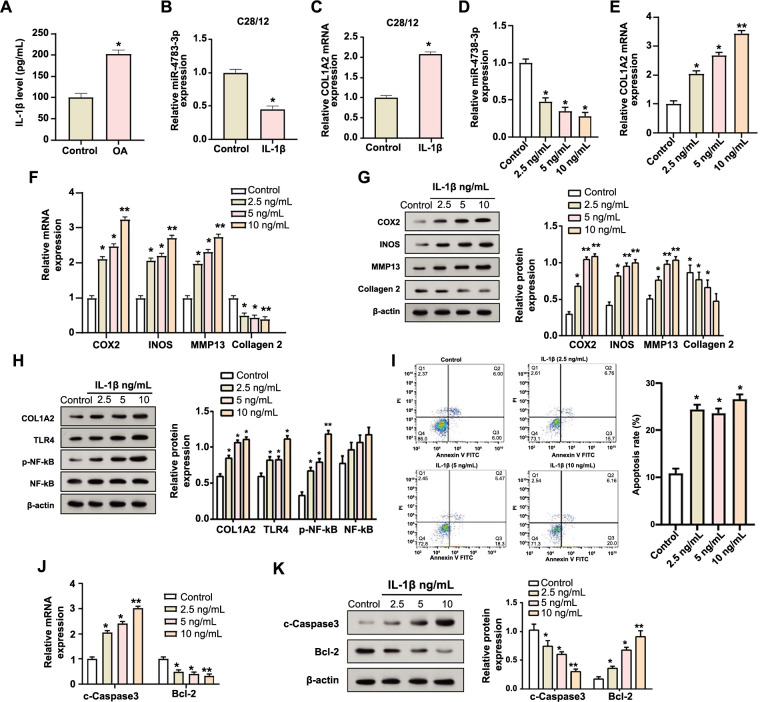
**Evaluation of IL-1β-induced changes in miR-4738-3p, *COL1A2* expression, and associated cellular responses in C28/I2 chondrocytes.** (A) Graph showing the relative IL-1β levels in C28/I2 chondrocytes after induction, confirming the establishment of the in vitro OA model. (B and C) qRT-PCR analysis depicting changes in the expression levels of miR-4738-3p (B) and *COL1A2* (C) in C28/I2 cells following IL-1β treatment. (D and E) qRT-PCR results showing dose-dependent effects of different concentrations of IL-1β on the expression of miR-4738-3p (D) and *COL1A2* (E), highlighting the significant changes at 10 ng/mL. (F and G) qRT-PCR (F) and WB (G) analyses depicting the differential expression patterns of chondrogenic markers induced by different IL-1β concentrations, including COX2, INOS, MMP13, and collagen 2. The accompanying bar graph on the right (G) displays protein expression quantification through gray-scale analysis. (H) WB analysis illustrating changes in protein levels of COLIA2, TLR4, p-NF-κB, and NF-κB at different doses of IL-1β. The accompanying bar graph on the right displays protein expression quantification through gray-scale analysis. (I) Flow cytometry results assessing the changes in apoptosis rate of C28/I2 chondrocytes after treatment with different IL-1β concentrations. (J and K) qRT-PCR (J) and WB (K) analyses demonstrating the differential expression trends of c-Caspase3 and Bcl-2 with increasing doses of IL-1β. The accompanying bar graph on the right (K) displays protein expression quantification through gray-scale analysis. *P < 0.05; **P < 0.01; indicating levels of statistical significance. IL-1β: Interleukin 1 beta; miR: MicroRNA; *COL1A2:* Collagen type I alpha 2 chain; OA: Osteoarthritis; qRT-PCR: Quantitative real-time polymerase chain reaction; WB: Western blot; COX2: Cyclooxigenase 2; INOS: Inducible nitric oxide synthase; MMP13: Matrix metallopeptidase 13; TLR4: Toll-like receptor 4; p-NF-κB: Phosphorylated nuclear factor kappa B; c-Caspase3: Cleaved-Caspase3; Bcl-2: B-cell lymphoma 2 protein; mRNA: Messenger RNA; PI: Propidium iodide; FITC: Fluorescein isothiocyanate.

We established an in vitro OA model by treating C28/I2 chondrocytes with IL-1β for 24 h. After induction, a notable upregulation of IL-1β levels was observed, confirming the establishment of our model (Figure 1A). Subsequent qRT-PCR analysis was conducted to assess the expression patterns of miR-4738-3p and *COL1A2* in the IL-1β-induced (10 ng/mL) C28/I2 chondrocytes. This analysis revealed a significant reduction in miR-4738-3p expression, while the *COL1A2* expression markedly increased following IL-1β treatment (Figure 1B and 1C). Moreover, dose-response studies indicated a dose-dependent impact on the expression levels of these molecules, with the 10 ng/mL IL-1β concentration showing the most pronounced effect (Figure 1D and 1E). Subsequent analysis, employing both qRT-PCR and WB techniques, revealed that chondrogenic markers, such as COX2, INOS, and MMP13, exhibited an upward trend in expression with increasing concentrations of IL-1β. In contrast, collagen 2 expression was observed to diminish (Figure 1F and 1G). Additionally, WB analyses demonstrated a dose-dependent surge in the protein levels of COL1A2. The role of TLR4 in mediating OA progression via the NF-κB signaling pathway and inflammatory cytokine production has been established in the literature [28]. In line with this understanding, our WB results underscored a dose-dependent augmentation in TLR4 and p-NF-κB protein levels, while the levels of NF-κB remained relatively unchanged across different IL-1β dosages (Figure 1H). Flow cytometry data further indicated an escalating trend in cell apoptosis rates corresponding to increasing IL-1β concentrations (Figure 1I). Consistently, qRT-PCR and WB assessments highlighted that c-Caspase3 expression levels increased and *Bcl-2* expression levels decreased with the rise in IL-1β concentrations (Figure 1J and 1K). Given these observations, particularly the pronounced changes at the 10 ng/mL IL-1β dosage, this concentration was selected for subsequent in-depth analyses.

### Characterization and differential expression analysis of miR-4738-3p and *COL1A2* in isolated exosomes and HS-MSC

**Figure 2.**
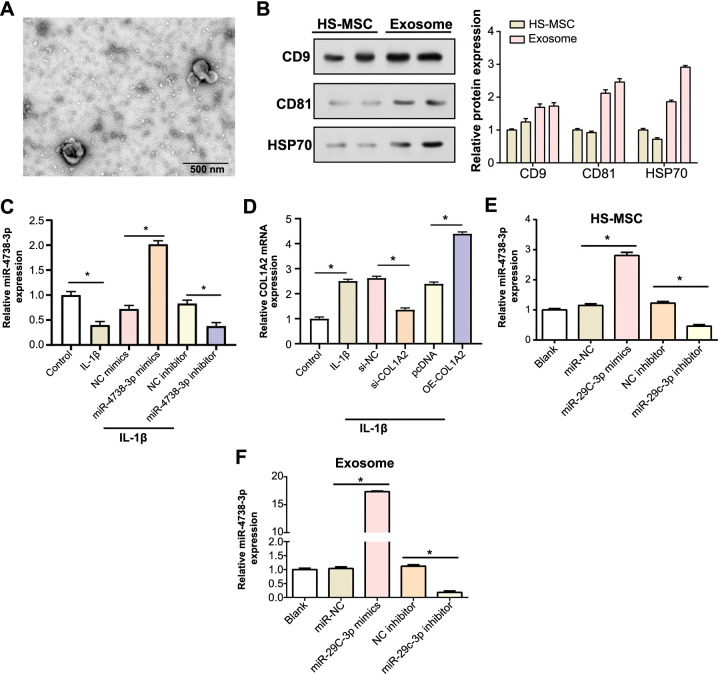
**Isolation of HS-MSC-exosomes and analysis of *COL1A2* and miR-4738-3p transfection efficiency.** (A) Transmission electron micrographs displaying the characteristic morphology of exosomes isolated from HS-MSC using ultracentrifugation. The scale bar indicates a length of 200 nm. (B) WB analysis detecting the protein levels of exosome markers (CD9, CD81, and HSP70) in both HS-MSCs and isolated exosomes. The accompanying bar graph on the right displays protein expression quantification through gray-scale analysis. (C and D) qRT-PCR analysis results revealing the expression trends of miR-4738-3p (C) and *COL1A2* (D) in IL-1β-induced C28/I2 chondrocytes. (E and F) Comparative qRT-PCR analysis results displaying the miR-4738-3p expression in HS-MSCs (E) and its derived exosomes (F). *P < 0.05; indicating the level of statistical significance. HS-MSC: Human synovial mesenchymal stem cells; *COL1A2:* Collagen type I alpha 2 chain; miR: MicroRNA; WB: Western blot; CD: Cluster of differentiation; HSP70: Heat shock protein 70; qRT-PCR: Quantitative real-time polymerase chain reaction; IL-1β: Interleukin 1 beta; NC: Negative control; mRNA: Messenger RNA; siRNA: Small interfering RNA; pcDNA: Empty vector control; OE: Overexpression vectors.

In this study, we utilized TEM to visualize and characterize exosomes isolated through ultracentrifugation. The TEM images delineated a population of vesicles exhibiting typical exosome morphology, characterized by well-preserved membrane integrity (Figure 2A). The observed size distribution of these vesicles fell within the typical exosome size range, thus affirming the successful isolation of exosomes. Subsequently, we embarked on understanding the protein composition of these exosomes in comparison to HS-MSC. WB analysis was utilized to assess the protein levels of well-known exosome markers, including CD9, CD81, and HSP70. The results unveiled significantly higher levels of these proteins in the exosomes as opposed to the HS-MSC, asserting the enriched protein content in the isolated exosomes (Figure 2B). To further delve into the functional aspects, we analyzed the expression levels of miR-4738-3p and *COL1A2* in IL-1β-induced C28/I2 cells using qRT-PCR. This analysis provided precise quantification of the expression alterations, displaying remarkable knockdown and overexpression efficiencies, thereby setting a strong premise for the role these molecules might be playing in the pathological context (Figure 2C and 2D). Expanding our investigation to include the expression profiles of miR-4738-3p in HS-MSC and exosomes, we observed significant overexpression of miR-4738-3p in the exosomes (Figure 2E and 2F). This finding suggests that miR-4738-3p is enriched in exosomes, highlighting their potential significance in exosome-mediated cell communication. This observation opens new avenues for exploring therapeutic interventions targeting these molecular pathways.

### Exo-miR-4738-3p alters expression profiles and inhibits apoptosis in an in vitro OA model

**Figure 3.**
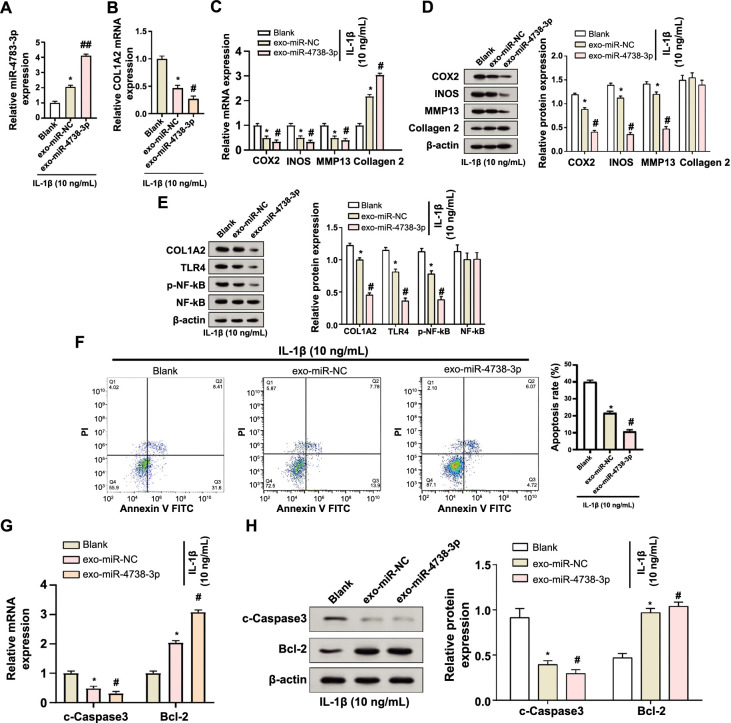
**Exo-miR-4738-3p alters the gene expression profile and inhibits apoptosis in OA chondrocytes.** (A and B) qRT-PCR analysis results displaying changes in the expression of miR-4738-3p (A) and *COL1A2* (B) in exosomes derived from C28/I2 chondrocytes following treatment with 10 ng/mL IL-1β. (C and D) qRT-PCR (C) and WB (D) assays measuring the expression levels of COX2, INOS, MMP13, and collagen 2 in the blank, exo-miR-NC, and exo-miR-4738-3p groups, following treatment with 10 ng/mL of IL-1β. The accompanying bar graph on the right (D) displays protein expression quantification through gray-scale analysis. (E) WB analysis demonstrating the protein levels of COL1A2, TLR4, p-NF-κB, and NF-κB in exosome-treated cells enriched with miR-4738-3p, following 10 ng/mL of IL-1β treatment. The accompanying bar graph on the right displays protein expression quantification through gray-scale analysis. (F) Flow cytometry presenting changes in cell apoptosis rates in the blank, exo-miR-NC, and exo-miR-4738-3p groups treated with 10 ng/mL of IL-1β. (G and H) Comparative qRT-PCR (G) and WB (H) analysis displaying the relative expression levels of c-Caspase3 and Bcl-2 in the blank, exo-miR-NC, and exo-miR-4738-3p groups, all based on 10 ng/mL of IL-1β treatment. The accompanying bar graph on the right (H) displays protein expression quantification through gray-scale analysis. *P < 0.05 vs the blank group; #*P* < 0.05 vs exo-miR-NC; indicating levels of statistical significance. Exo: Exosome; miR: MicroRNA: OA: Osteoarthritis; qRT-PCR: Quantitative real-time polymerase chain reaction; *COL1A2:* Collagen type I alpha 2 chain; IL-1β: Interleukin 1 beta; WB: Western blot; COX2: Cyclooxigenase 2; INOS: Inducible nitric oxide synthase; MMP13: Matrix metallopeptidase 13; NC: Negative control; TLR4: Toll-like receptor 4; p-NF-κB: Phosphorylated nuclear factor kappa B; c-Caspase3: Cleaved-Caspase3; Bcl-2: B-cell lymphoma 2 protein; mRNA: Messenger RNA; PI: Propidium iodide; FITC: Fluorescein isothiocyanate.

Following exposure to 10 ng/mL of IL-1β for 24 h, qRT-PCR analyses of the exosomes derived from HS-MSCs revealed a significant upregulation in the expression of miR-4738-3p. Concurrently, a notable decrease in the expression of *COL1A2* was observed (Figure 3A and 3B). These exosomes, enriched with exo-miR-4738-3p, underwent comprehensive analysis using both qRT-PCR and WB assays. Intriguingly, there was a marked downregulation in the expression levels of pro-inflammatory markers, including COX2, INOS, and MMP13. Conversely, collagen 2, an essential component of the cartilage matrix, showed a pronounced upregulation (Figure 3C and 3D). Further WB analyses provided deeper insights into the effects of exo-miR-4738-3p enrichment, revealing a significant reduction in the expression levels of COL1A2, TLR4, and p-NF-κB in miR-4738-3p-enriched exosomes compared to controls (Figure 3E). Flow cytometry analyses shed light on the apoptosis rates, revealing that cells treated with exo-miR-4738-3p exhibited lower apoptosis rates in comparison to the control group (Figure 3F). Supporting these findings, qRT-PCR and WB analysis revealed a downward trend in c-Caspase3 expression, a pivotal executor of apoptosis, and an upregulation in the expression of the anti-apoptotic marker Bcl-2 (Figure 3G and 3H). Collectively, these findings suggest a potential protective role of exo-miR-4738-3p in chondrocytes under OA conditions. This implies a modulation in the inflammatory response and a shift toward enhanced cellular survival.

### Knockdown of miR-4738-3p exacerbates IL-1β-induced damage in C28/I2 chondrocytes

**Figure 4.**
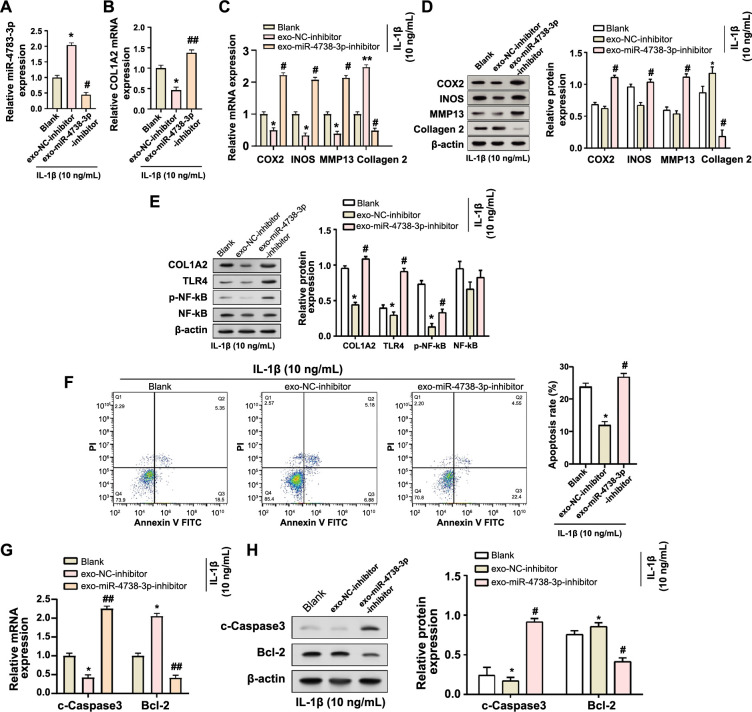
**Knockdown of exo-miR-4738-3p exacerbates IL-1β-induced C28/I2 chondrocyte injury.** (A and B) qRT-PCR verifying the knockdown efficiency of miR-4738-3p (A) and illustrating the corresponding changes in *COL1A2* expression (B) in exosomes derived from C28/I2 chondrocyte treated with 10 ng/mL of IL-1β. (C and D) qRT-PCR (C) and WB (D) assays measuring the expression levels of COX2, INOS, MMP13, and collagen 2 in the blank, exo-NC-inhibitor, and exo-miR-4738-3p inhibitor groups following treatment with 10 ng/mL of IL-1β. The accompanying bar graph on the right (D) displays protein expression quantification through gray-scale analysis. (E) WB analysis demonstrating the protein levels of COL1A2, TLR4, p-NF-κB, and NF-κB in exosome-treated cells enriched with the miR-4738-3p inhibitor, following 10 ng/mL of IL-1β treatment. The accompanying bar graph on the right displays protein expression quantification through gray-scale analysis. (F) Flow cytometry presenting changes in cell apoptosis rates in blank, exo-NC-inhibitor, and exo-miR-4738-3p inhibitor groups treated with 10 ng/mL of IL-1β. (G and H) Comparative qRT-PCR (G) and WB (H) analysis displaying the relative expression levels of c-Caspase3 and Bcl-2 in the blank, exo-miR-NC, and exo-miR-4738-3p groups, all based on 10 ng/mL of IL-1β treatment. The accompanying bar graph on the right (H) displays protein expression quantification through gray-scale analysis. *P < 0.05 vs the blank group; **P < 0.01 vs the blank group; #*P* < 0.05 vs exo-miR-NC; indicating levels of statistical significance. Exo: Exosome; miR: MicroRNA; IL-1β: Interleukin 1 beta; qRT-PCR: Quantitative real-time polymerase chain reaction; *COL1A2:* Collagen type I alpha 2 chain; WB: Western blot; COX2: Cyclooxigenase 2; INOS: Inducible nitric oxide synthase; MMP13: Matrix metallopeptidase 13; NC: Negative control; TLR4: Toll-like receptor 4; p-NF-κB: Phosphorylated nuclear factor kappa B; c-Caspase3: Cleaved-Caspase3; Bcl-2: B-cell lymphoma 2 protein; mRNA: Messenger RNA; PI: Propidium iodide; FITC: Fluorescein isothiocyanate.

We further investigated the effects of miR-4738-3p knockdown in the exosomes derived from C28/I2 chondrocytes treated with IL-1β. The knockdown efficiency was validated through qRT-PCR, which demonstrated a significant downregulation in the expression of miR-4738-3p, following the knockdown. Conversely, an upregulation in the expression levels of *COL1A2* was observed (Figure 4A and 4B). Subsequent analyses employing both qRT-PCR and WB techniques revealed a differential expression pattern of several crucial markers following miR-4738-3p knockdown. Notably, there was a significant increase in the levels of COX2, INOS, and MMP13, all typically associated with pro-inflammatory responses. Conversely, the expression of collagen 2, a vital marker for cartilage integrity, was found to be downregulated (Figure 4C and 4D). Additionally, further WB analyses displayed a corresponding upregulation in COL1A2, TLR4, and p-NF-κB levels, indicating a heightened inflammatory response following miR-4738-3p knockdown (Figure 4E). Finally, we evaluated the apoptosis rates following the knockdown. A pronounced increase in the apoptosis rates was evident, demonstrating the detrimental effects of miR-4738-3p knockdown (Figure 4F). Consistent with this observation, elevated levels of c-Caspase3, a known apoptosis facilitator, alongside reduced expression of Bcl-2, a well-established anti-apoptotic marker, were observed. This finding corroborated the intensified cell damage under IL-1β induction post-miR-4738-3p knockdown (Figure 4G and 4H). Collectively, these findings strongly suggest that the knockdown of miR-4738-3p exacerbates IL-1β-induced damage in C28/I2 chondrocytes, highlighting the crucial protective role of miR-4738-3p in this cellular context.

### miR-4738-3p regulates *COL1A2* expression through NF-κB signaling pathway modulation

**Figure 5.**
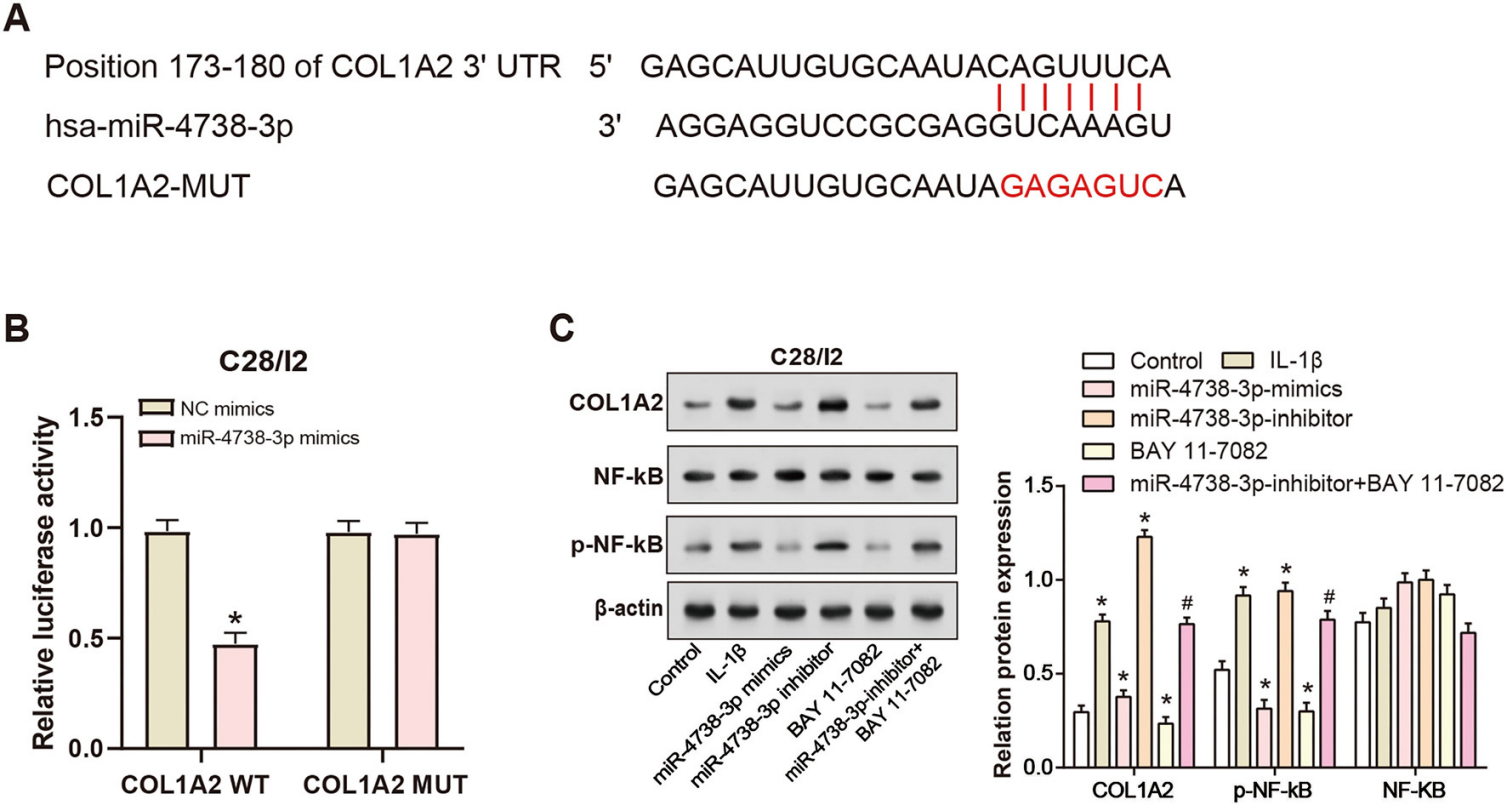
**miR-4738-3p regulates the NF-κB signaling pathway by targeting *COL1A2*.** (A) Schematic representation from the ENCORI database, depicting the potential binding sites between miR-4738-3p and the 3′-UTR of *COL1A2*. (B) Dual-luciferase reporter assay results confirming the direct interaction between miR-4738-3p and the 3′-UTR of *COL1A2*. *P < 0.05; indicating levels of statistical significance. (C) WB analyses depicting the effect of various treatments (control, Il-1β, miR-4738-3p mimics, miR-4738-3p inhibitor, BAY-11-7082, miR-4738-3p inhibitor + BAY 11-7082) on the expression levels of COL1A2 and p-NF-κB, highlighting the regulatory role of miR-4738-3p in the NF-κB signaling pathway. *P < 0.05 indicating a significant difference compared to the control group. ^#^*P* < 0.05 indicating a significant difference between miR-4738-3p-inhibitor + BAY 11-7082 group and the miR-4738-3p-inibitor group or BAY 11-7082 group. The accompanying bar graph on the right displays protein expression quantification through gray-scale analysis. miR: MicroRNA; NF-κB: Nuclear factor kappa B; *COL1A2*: Collagen type I alpha 2 chain; ENCORI: Encyclopedia of RNA Interactomes; 3′-UTR: 3′-untranslated region; WB: Western blot; IL-1β: Interleukin 1 beta; p-NF-κB: Phosphorylated nuclear factor kappa B; hsa: *Homo sapiens*; MUT: Mutation; NC: Negative control.

Utilizing the Encyclopedia of RNA Interactomes (ENCORI) database, we identified potential binding sites between miR-4738-3p and the 3′-UTR of *COL1A2*, suggesting a potential post-transcriptional regulatory relationship (Figure 5A). To validate this interaction, a dual-luciferase reporter assay was employed. The results of this assay unveiled that miR-4738-3p mimics significantly attenuated the luciferase activity of the *COL1A2*-wild type (wt) 3′-UTR, reinforcing the notion of direct interaction between miR-4738-3p and the 3′-UTR of *COL1A2* (Figure 5B). To further investigate the underlying mechanisms, BAY-11-7082, a recognized inhibitor of the NF-κB signaling pathway that blocks NF-κB phosphorylation, was introduced. WB analyses illustrated that treatment with either IL-1β or miR-4738-3p inhibitor led to an upregulation in the levels of *COL1A2* and p-NF-κB. Conversely, the introduction of miR-4738-3p mimics or treatment with BAY-11-7082 resulted in a marked downregulation of both *COL1A2* and p-NF-κB levels. Interestingly, co-treatment with the miR-4738-3p inhibitor and BAY-11-7082 partially restored the levels of *COL1A2* and p-NF-κB. These findings collectively indicate that miR-4738-3p regulates *COL1A2* expression, potentially through the modulation of the NF-κB signaling pathway. The observed partial recovery in *COL1A2* and p-NF-κB levels upon co-treatment with the miR-4738-3p inhibitor and BAY-11-7082 further highlights the interconnected regulatory role of miR-4738-3p within this pathway (Figure 5C).

## Discussion

OA is a debilitating joint disease characterized by progressive articular cartilage degeneration and subchondral bone remodeling [29, 30]. The knee joint is frequently affected in OA, with specific attention given to the infrapatellar fat pad (IPFP), also known as Hoffa’s fat pad. Functionally, the IFP is thought to primarily absorb forces generated during knee motion. Microarray analysis has revealed a significant upregulation of genes associated with fat metabolism and energy homeostasis in the knee IFP of patients with end-stage OA [31]. Furthermore, it has been reported that in the context of OA, IFP-derived stem cells may undergo reprogramming and activation in response to the inflammatory milieu, retaining their multidifferentiation plasticity [32]. Prior histological studies have detailed the degenerative changes in the meniscus, including detachment of fibrocartilage from the stroma, abrasion, lacerations, calcification, and the presence of diffuse cellular hyperplasia, hypertrophy, and abnormal cell clusters within the meniscal matrix [33]. Additionally, in recent studies, advanced stages of OA in the knee have been shown to exhibit severe collagen fiber disorganization accompanied by an increase in proteoglycans, as observed through electron microscopy and histological examinations [34]. These findings collectively highlight the structural joint changes that occur during OA progression, underscoring the complex pathology involving alterations in both articular cartilage and bone tissue. To elucidate the molecular dynamics of OA, we conducted a detailed analysis of the GSE55235 dataset. Central to our findings is the identification of the green module through the WGCNA method approach. This module showed a significant association with the OA phenotype, underscoring the relevance of gene networks in understanding the complexities of OA pathogenesis. Notably, the overlapping genes and DEGs within this green module were identified as potentially crucial in the progression of OA. Among these, *COL1A2* emerged as a hub gene with prominent expression in C28/I2 cells, indicating its potential central role in the molecular cascade of OA. Additionally, the GEPIA database confirmed a clear negative correlation between miR-4738-3p and *COL1A2*. This finding raises pertinent questions regarding their interaction in OA pathogenesis.

The critical role of IL-1β in modulating molecular pathways and promoting apoptosis in the context of OA is evident from the presented data and the broader literature [35, 36]. Our results in the C28/I2 chondrocyte model accentuate this role, especially highlighted by the significant upregulation of IL-1β levels and its subsequent downstream molecular effects. The concomitant decrease in miR-4738-3p with an increase in *COL1A2* expression highlights a complex molecular interplay instigated by IL-1β induction. Various studies have confirmed IL-1β as an impetus in the pathogenesis of OA, emphasizing its capability to modify gene expressions, particularly those related to apoptosis and cartilage integrity [37, 38]. For instance, markers of chondrogenesis that play a key role in cartilage degradation, such as COX2, INOS, and MMP13 [39], are elevated in response to IL-1β. Interestingly, collagen 2, which is essential for cartilage structure and function [40], underwent downregulation, further revealing an environment of OA deterioration driven by IL-1β. Additionally, dose-dependent increases in TLR4 and p-NF-κB following IL-1β induction reaffirm the contribution of the TLR4-mediated NF-κB signaling pathway in OA progression, specifically through the production of inflammatory cytokines. The notable changes observed at the 10 ng/mL IL-1β concentration align with findings from other studies, validating the efficacy of our in vitro OA model. The significant role of IL-1β in inducing apoptosis, marked by increased c-Caspase3 and decreased Bcl-2 levels, aligns with its recognized adverse impact in OA, thereby suggesting potential therapeutic targets for disease management. These findings on cartilage-related proteins emphasize the necessity for targeted therapeutics and further mechanistic investigations in OA research.

Exosomes, submicron-sized vesicles derived from endosomal compartments, have recently emerged as key players in intercellular communication [41, 42]. These vesicles, secreted by various cell types including MSCs, serve as carriers of molecular information (proteins, lipids, and nucleic acids) [43, 44]. Their ability to replicate some therapeutic benefits of parent cells makes them particularly valuable in regenerative medicine [45]. In recent years, the prospect of HS-MSC-derived exosomes in disease regulation has garnered attention. For instance, in a comprehensive review, Kim et al. outlined the potential of MSC-derived exosomes in OA treatment, emphasizing their ability to reduce inflammation, promote cartilage extracellular matrix (ECM) expression, and enhance cartilage tissue regeneration, while overcoming some of the limitations associated with direct MSC-based therapies [46]. Another study also showed that IPFP MSC-derived exosomes could regulate cartilage homeostasis and improve gait abnormalities in mice through miR100-5p-mediated inhibition of the mechanistic target of rapamycin (mTOR) autophagy pathway. This suggests that IPFP MSC-derived exosomes may become a potential therapy in OA treatment [47]. The importance of miRNAs within these exosomes has also been recognized. As small, non-coding RNA molecules, miRNAs play a key role in post-transcriptional gene regulation [48]. Their role in OA pathogenesis is increasingly evident, as highlighted by studies such as the one conducted by Wu et al. This particular study demonstrated that the exosomal delivery of miR-140 could significantly impact targeted OA therapy. Such findings contribute to the evolving paradigm of cell-free therapy, significantly shaping our current understanding of OA and its potential treatment approaches [49]. MiR-486-5p modified exosomes, for example, have been shown to effectively alleviate chondrocyte apoptosis and OA, showing superior results compared to direct miR-486-5p administration, offering a promising miRNA-based therapeutic strategy for OA [50]. Similarly, Zhou et al. [51] found that synovial fluid-derived exosomal miRNA-126-3p was reduced in OA patients and could inhibit chondrocyte inflammation and prevent cartilage degeneration. Consistent with this, our results reveal subtle interactions between exosomes, miR-4738-3p, and *COL1A2* in OA. Our data demonstrate that exosomes not only increase the expression of miR-4738-3p and *COL1A2* but also play a role in regulating apoptosis and cell damage. This is evident from molecular changes observed in markers, such as COX2, INOS, MMP13, and TLR4. Particularly, significant alterations in apoptotic markers, notably c-Caspase3 and Bcl-2, were observed following the modulation of miR-4738-3p levels. These findings highlight the therapeutic potential of exosomes, especially in how the interplay of miR-4738-3p, *COL1A2*, and exosomes can modulate inflammatory responses, potentially enhancing cell survival and tissue regeneration in OA.

Intracellular signaling cascades play a critical role in regulating numerous processes that determine cell fate, especially under pathological conditions [52]. Complex networks of miRNAs, serving as master regulators of gene expression, often interact with these signaling pathways, adding another layer of complexity [53]. Our current findings contribute to this narrative by elucidating the interactions of miR-4738-3p, *COL1A2*, and NF-κB signaling pathways. Data from ENCORI confirm the existence of a potential binding site between miR-4738-3p and *COL1A2* 3′-UTR. Providing tangible evidence of a direct post-transcriptional regulatory association, our validation using a dual-luciferase reporter assay demonstrated that miR-4738-3p effectively reduces the luciferase activity of the *COL1A2*-wt 3′-UTR, illustrating a nuanced regulatory mechanism by which miR-4738-3p regulates *COL1A2* expression. The NF-κB signaling pathway is a classic signaling cascade intricately linked to inflammation, cell survival, and other important biological processes [54, 55]. Previous studies have found that the abnormal activation of the NF-κB pathway is associated with various pathologies, including OA. For example, the NF-κB signaling pathway is known to be overactivated in OA, and this heightened activation is associated with disease progression. It contributes to the deterioration of cartilage and promotes the production of pro-inflammatory cytokines, key factors in the advancement of OA [56, 57]. In addition, studies by Guo et al. [58] have identified the role of NF-κB in synovial cell activation and chondrocyte apoptosis. Studies have also shown that anthocyanins exert anti-inflammatory effects in OA by inhibiting the sirtuin 6 (Sirt6)/NF-κB axis [59]. Ding et al. [60] demonstrated that in OA, miR-93 can target and inhibit TLR4, a key regulator of the NF-κB signaling pathway, thereby reducing chondrocyte apoptosis and inflammation. In our observations, the relationship between miR-4738-3p, *COL1A2*, and NF-κB was found to be critical. The observed modulation of *COL1A2* and p-NF-κB levels in response to treatments with miR-4738-3p mimics, inhibitors, and NF-κB pathway inhibitor BAY-11-7082 emphasizes the regulatory role of miR-4738-3p. Notably, the partial recovery of *COL1A2* and p-NF-κB levels following combined treatment highlights the complex and interconnected effects of miR-4738-3p on this pathway. Taken together, the dynamic crosstalk between miR-4738-3p and *COL1A2*, coupled with the regulation of the NF-κB signaling pathway, provides new insights into the complex molecular mechanisms of OA. Understanding these intricate relationships is crucial not only for unraveling complex regulatory networks but also for highlighting potential therapeutic targets. Such insights could pave the way for the development of more effective intervention strategies in OA.

## Conclusion

In this study, we reveal a novel regulatory axis in OA pathophysiology, involving exosomes, miR-4738-3p, *COL1A2*, and NF-κB signaling pathways. Our findings indicate that exosomes serve as important carriers of miR-4738-3p, which regulates *COL1A2* expression post-transcriptionally. Interestingly, this regulatory effect is further mediated through the modulation of the NF-κB signaling cascade. Such regulation through the NF-κB signaling pathway appears to contribute to the regulation of OA-associated inflammation and cellular damage. This complex molecular interaction opens up new therapeutic possibilities, highlighting the potential of exosome-mediated delivery of miR-4738-3p as a novel strategy for mitigating OA inflammation and enhancing cellular health.

## Supplemental data

**Table S1:** Primer sequences of qRT-PCR used in this study

**Gene symbol**	**Forward primer (5′ – 3′)**	**Reverse primer (5′ – 3′)**
miR-4738-3p	TGAAACTGGAGCGCCTGG	CAGTGCGTGTCGTGGAGT
*COL1A1*	GAGTCACACCGGAGCCTGGGG	GCATGGGTCTTCAAGCAAGTG
*COL1A2*	CATCCCAGCCAAGAACTGGTA	GACTGGGCCAATGTCCACAAA
*COL3A1*	CCCGTATTATGGAGATGAAC	TCAGGACTAATGAGGCTTTCT
*COL5A1*	AAGCGTGGGAAACTGCTCTCCTAT	AGCAGTTGTAGGTGACGTTCTGGT
*COL5A2*	CAGGCTCCATAGGAATCAGAGG	CCAGCATTTCCTGCTTCTCCAG
*COX2*	GCTGGAACATGGAATTACCCA	CTTTCTGTACTGCGGGTGGAA
*INOS*	AGACCTCAACAGAGCCCTCA	GCAGCCTCTTGTCTTTGACC
*MMP13*	GCACTTCCCACAGTGCCTAT	AGTTCTTCCCTTGATGGCCG
Collagen 2	CCTACAATAATAATATATACCCCACCA	ATGTGTTTTCAGTGATCATGTTTTC
c-Caspase3	ACAGTGGAACTGACGATGATATG	TCCCTTGAATTTCTCCAGGAATAG
*Bcl-2*	GTGGATGACTGAGTACCTGAAC	GAGACAGCCAGGAGAAATCAA
U6	CTCGCTTCGGCAGCACATATACT	ACGCTTCACGAATTTGCGTGTC
*GAPDH*	CCACTCCTCCACCTTTG	CACCACCCTGTTGCTGT

qRT-PCR: Quantitative real-time polymerase chain reaction; miR: MicroRNA; *COL1A1*: Collagen type I alpha 1 chain; *COL1A2*: Collagen type I alpha 2 chain; *COL3A1*: Collagen type III alpha 1 chain; *COL5A1*: Collagen type V alpha 1 chain; *COL5A2*: Collagen type V alpha 2 chain; *COX2*: Cyclooxigenase 2; *INOS*: Inducible nitric oxide synthase; *MMP13*: Matrix metallopeptidase 13; c-Caspase3: Cleaved-Caspase3; *Bcl2*: B-cell lymphoma 2; U6: U6 small nuclear RNA; *GAPDH*: Glyceraldehyde 3-phosphate dehydrogenase.

## Data Availability

The datasets used and/or analyzed during the current study are available from the corresponding author upon reasonable request.
